# Response Inhibition as a Function of Movement Complexity and Movement Type Selection

**DOI:** 10.3389/fpsyg.2018.02290

**Published:** 2018-11-26

**Authors:** Germán Gálvez-García, Javier Albayay, Lucio Rehbein, Claudio Bascour-Sandoval, George A. Michael

**Affiliations:** ^1^Departamento de Psicología, Universidad de La Frontera, Temuco, Chile; ^2^Département de Psychologie Cognitive, Sciences Cognitives et Neuropsychologie, Institut de Psychologie, Laboratoire d’Étude des Mécanismes Cognitifs, Université Lyon 2, Lyon, France; ^3^Dipartimento di Psicologia Generale, Università degli Studi di Padova, Padova, Italy; ^4^Carrera de Kinesiología, Facultad de Ciencias de la Salud, Universidad Autónoma de Chile, Temuco, Chile

**Keywords:** response inhibition, motor complexity, Simon effect, delta-plots, kinematic errors

## Abstract

This study aims to determine whether response inhibition shows the same degree of effectiveness for two sources of motor complexity: (1) Movement complexity, which is measured through two actions with different motor requirements (simple lifting action vs. complex reaching action), and (2) Movement type selection, which is measured in movements performed separately (no active-movement type selection) vs. selectively (active-movement type selection). Activation–suppression model was tested in three experiments to measure activation of the preponderant responses and subsequent suppression in a Simon task. More errors and higher magnitude of congruence effect (which reflects greater effectiveness of response suppression) were expected for more difficult motor conditions. Reaction time, movement time, kinematic errors, and movement errors were recorded. Results of Experiment 1, in which movement type selection was not active, showed that both movements did not differ in their activation and suppression, as they presented similar kinematic error rates and Simon effects. Experiment 2, in which movement type selection was active, resulted in a higher kinematic error rate and higher magnitude of Simon effect in lifting. These results were confirmed in Experiment 3, in which participants performed all experimental motor complexity conditions. Finally, Experiment 4 showed that responses with similar movement complexity did not differ in their activation and suppression, even when movement type selection was active. Thus, the present study provides evidence on the varying effectiveness of response inhibition as a function of movement complexity, but only in demanding situations in which movement type selection is active. These results can be attributed to a top-down strategy to minimize error for actions most prone to develop kinematic error.

## Introduction

Response inhibition is among the key executive functions in daily life to stop unwanted and incorrect motor actions (Diamond, 2013). Most behavioral and neuroscientific studies have focused on neural substrates (e.g., Rubia et al., 2001; Stelzel et al., 2006; Li et al., 2008; Levy and Wagner, 2011) and the underlying mechanisms on response inhibition (De Jong et al., 1995; Logan et al., 1997). However, not all actions are equivalent with respect to their motor complexity and the degree of control that exists within their underlying movements (Henry and Rogers, 1960; Ma and Trombly, 2004). Although neuroimaging studies have consistently shown an increase in activation in many areas of the cortex, such as sensorimotor cortex, as a function of motor complexity (Rao et al., 1993; Shibasaki et al., 1993; Wexler et al., 1997; Verstynen et al., 2005), few studies have tested directly the relation between response inhibition and motor complexity.

Response selection studies have reached different conclusions. For example, Pratt et al. (2014) studied the impact of relative motor load in response inhibition of children with developmental coordination disorder compared with a control group. Inhibition was assessed by the Stroop task (Stroop, 1935; low motor load) and NEPSY Tower task (Korkman et al., 1998; high motor load). They hypothesized a diminished response inhibition in high motor load based on previous literature, which demonstrated a significant relationship between motor abilities and response inhibition (i.e., better motor performance with more proficient inhibition; Livesey et al., 2006). In general and contrary to predictions, the level of motor load did not seem to affect task performance.

Meanwhile, studies on motor complexity and switch cost (for a review of this effect, see Monsell, 2003) have found evidence on the impact of motor complexity in response inhibition. For example, Gálvez-García et al. (2018) found that sequential complex actions (use of different hands in two actions performed in sequence) showed switching costs (long latencies to switch from one effector to another) compared with simpler actions (use the same hand in two actions performed in sequence). This effect was partially attributed to stronger motor interhemispheric inhibition in complex actions (Trapp et al., 2012) (see Hübner and Druey, 2006; Koch et al., 2010 for a revision of response inhibition in task switching). Similar results were found by Greenhouse et al. (2015) in studying the effect of motor complexity in preparatory inhibition. Greenhouse et al. (2015) compared single index finger response with a more complex response where two fingers from the same hand required a coordinated gesture in a choice reaction time task. Larger preparatory inhibition (i.e., slower reaction times and more suppressed motor-evoked potentials) was found in complex movements, especially from the non-selected hand. Greenhouse et al. (2015) concluded that recruitment of lateral prefrontal cortex (the brain region especially sensitive to response complexity in response selection, Botvinick et al., 2001; Ridderinkhof et al., 2002a) would be greater along the preparation of complex movements to minimize response selection and execution errors. Moreover, an additional inhibition mechanism of the selected response associate with impulsive control was proposed (Hasbroucq et al., 1999a,b; Duque et al., 2012). However, error rate and subsequent analyses have not been reported, probably because the task is not demanding, making it difficult to establish conclusions on the impact of motor complexity on impulse control of the action and the influence the level of excitability for the responses.

As Houghton et al. (1996) highlighted, two mechanisms act together in selective attention and response inhibition: activation of the preponderant responses and its subsequent inhibition. However, activation–suppression models in cognitive control, such as conflict monitoring hypothesis (Botvinick et al., 2001), activation suppression model (Ridderinkhof, 2002a,b), and neural network model in selective attention (Houghton et al., 1996), have not proposed direct predictions with respect to motor complexity and response inhibition. In view of this, the aim of the present study was to determine the extent to which motor complexity impacts response inhibition and its components, For this aim, we tested the activation–suppression model postulated by Ridderinkhof (2002a,b) and a related congruence effect associated with this model, namely, the Simon effect.

### Simon Effect and Activation–Suppression Model

The Simon effect (Simon and Rudell, 1967) relates to a conflict paradigm widely used to study response inhibition (for a revision, see Ridderinkhof et al., 2002a). The most common observation is that responses are faster when stimuli are presented on the same side as the hand used to respond (i.e., congruent trials) than when they are presented on the opposite side (i.e., incongruent trials). According to the dual-route model (De Jong et al., 1994), a direct route processes the irrelevant spatial dimension, triggering a quick and automatic congruent response, whereas a controlled route processes the relevant color or shape of the target, activating the response assigned under instructions. Accordingly, the Simon effect occurs owing to a conflict produced by the joint activation of both routes during incongruent trials (Burle et al., 2002). Consequently, the automatic response triggered through the direct route must be inhibited. This effect renders a significant influence in the response selection stage (Kornblum, 1994; Rubichi and Pellicano, 2004). Several authors have studied its features regarding response inhibition (e.g., De Jong et al., 1994; Ridderinkhof, 2002a; Burle et al., 2013). They postulated that suppression in selective response inhibition can be examined based on the reaction time distribution of the Simon task using delta-plots, whereas initial response activation is represented through the delta-plots of accuracy (e.g., Burle et al., 2002; Buetti and Kerzel, 2008; Pratte et al., 2010; Wylie et al., 2010; Proctor et al., 2011; Dittrich et al., 2014; Suarez et al., 2014; Duprez et al., 2016; Miller and Roüast, 2016). Delta-plots of response suppression are generated by plotting the magnitude of the Simon effect (i.e., the difference between incongruent and congruent trials) as a function of response speed (Burle et al., 2013), allowing the visualization of the magnitude of response suppression (i.e., more or less effective). In a classical Simon task, delta-plots show the initial increase of the effect size through a positive slope, followed by a leveling off and decay of the Simon effect as reaction time increases (De Jong et al., 1994; Ridderinkhof et al., 2002a, 2005). Delta-plots of response activation (i.e., accuracy) are generated by the error rate of incongruent trials in which more errors are expected on the faster incongruent trials (Wylie et al., 2010), especially in conditions with a less effective inhibition (Ridderinkhof et al., 2005). According to activation–suppression model, automatic responses activated through the direct route are selectively inhibited, and thus, selective response suppression requires some time to build up and reach effectiveness. Here, the conflict described by the dual-route model (De Jong et al., 1994) is resolved by the suppression of incorrect responses during incongruent trials. Following this logic, the more effective the suppression, the more negative the slope of the delta-plot, thereby more often preventing the manifestation of inappropriate incongruent responses.

Certain predictions could be taken from activation–suppression model regarding motor complexity. As more complex motor demands present more errors (Henry and Rogers, 1960; Hazeltine et al., 2002; Monsell, 2003; Ma and Trombly, 2004), a subsequent and more effective inhibition could be reflected in the suppression of incorrect responses in actions with higher motor complexity, in agreement with the idea that inhibitory control increases after errors in the Simon task as a mechanism of cognitive control (i.e., reduction of impulsive error responses). In addition, more complex motor demands would allow more time to build selective response suppression and reach greater effectiveness. Thus, it would be expected to find smaller Simon effects magnitude (i.e., more effective response suppression) in actions with higher motor demands owing to the (a) high error rates accompanied by a subsequent greater response suppression and (b) slower reaction time that would allow this process to become more effective.

### Present Study

To test our prediction about activation–suppression model and motor complexity, we developed a series of experiments in which two sources of motor complexity are studied: movement complexity and movement type selection. For the manipulation of movement complexity (Experiment 1), we chose two movements with different requirements: reaching and lifting. Differences between movements are limited to a greater number of motor parameters to be programmed in the reaching action (Henry and Rogers, 1960; Ma and Trombly, 2004). In terms of the number of involved muscles, the lifting action mainly requires the activation of the common extensor muscle of the fingers (i.e., extensor digitorum muscle), and the extensor muscle of index finger (i.e., extensor indicis muscle), whereas the reaching action involves the activation of other muscles (pectoral and shoulder muscles) (Buneo et al., 1994). Both movements are performed separately in different experimental blocks. However, several tasks are not restricted to choosing the correct effector (i.e., left or right) but also requires choosing the proper motor action. Subsequently, it is unclear how the aforementioned response inhibition works when the motor actions are executed alternately, as is usual in everyday life. We referred to this as *movement type selection*. For the manipulation of movement type selection, we compared the results of Experiment 1, in which two movements with different motor complexity levels (i.e., a simple action vs. a more complex action) are performed in isolation (no active-movement type selection), with Experiment 2, in which reaching and lifting are performed alternately in the same experimental block, including the cognitive operation of action selection (active-movement type selection). As far as movement complexity is concerned, we hypothesize that the more complex movement would show a smaller Simon effect (reflected in a more negative-going slope in the delta-plots) owing to the higher error rate and slower latencies. This tendency would indicate that complex movements are inhibited more effectively. Further, the hypothesized differences could be more pronounced when action selection is required (i.e., active-movement type selection) because of the extra process needed to choose the proper action (i.e., the motor effector and the motor action have to be selected) and the subsequent likelihood to perform more errors.

The interaction between movement type selection conditions was directly assessed in Experiment 3 to rule out individual differences between participants as an explanatory factor. Finally, the comparability of similar movement complexity between the actions was assessed in Experiment 4 to determine its impact on response inhibition.

## Experiment 1: No Active-Movement Type Selection

### Methods

#### Participants

The sample consisted of 16 right-handed participants (seven males; mean age 22.25 ± 3.02 years) with normal or corrected-to-normal vision. This study was carried out in accordance with the recommendations of the ethical committee from the University of La Frontera. All subjects gave written informed consent in accordance with the Declaration of Helsinki. The protocol was approved by the same ethical committee.

#### Apparatus and Stimuli

**FIGURE 1:**
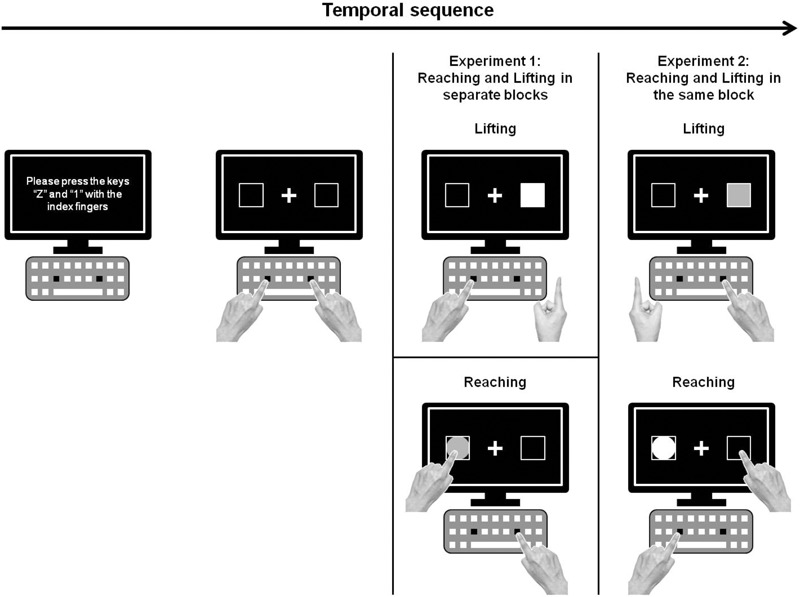
Schematic drawing of the experimental set up and procedure of Experiments 1 and 2 (green stimuli are presented as white, and red stimuli, as gray).

The stimuli were presented on a 17-inch touch screen, placed at a distance of 15 cm from a computer keyboard (see Figure 1). Participants placed their hands on the keyboard and then pressed two keys with their index fingers (“Z” with the left and “1” on the numeric keypad with the right) to start each experimental trial. Stimuli consisted of a circle (5 cm diameter) or square (5 cm × 5 cm area) prompts of green or red color, placed in the center of one of two boxes (5 cm × 5 cm area; black color with a white edge 10 mm in width), at both external lateral sides of the computer screen at 4 cm from the edge. A white fixation cross (0.13° of visual angle) was presented in the center of the touch screen against a black background. Time programming and data collection for the experimental conditions were carried out using Presentation software (Neurobehavioral System). The beginning of the movements (i.e., release for lifting and reaching actions) was measured by the release mechanism of Presentation software. The end of movement for reaching was registered using a tactile screen (Temporal sampling rate of 100 Hz), which had to be touched by the participant. The experiment was conducted in a dimly lit room.

#### Procedure

Two experimental blocks composed Experiment 1: responding by lifting in one block and by reaching in the other. The event sequencing of each experimental trial (see Figure 1) started with a screen that instructed participants to place their index fingers on the assigned keyboard keys (“Z” and “1” for the left and right hands, respectively). After a 1,000-ms interval, a centered white fixation cross and two lateralized boxes appeared on the screen against a black background. After an interval ranging from 1,000 to 2,000 ms (random inter-stimulus interval), the target stimulus appeared randomly and equiprobably in the center of one of the boxes (left or right). The target stimulus remained until the subject’s response. Auditory feedback (a 400-Hz computer-generated tone for 100 ms) was provided after error trials. Subjects were required to respond according to the color of the stimulus, not location. When the stimulus was red, participants had to respond with their right hand, whereas when the stimulus was green, they had to respond using their left hand. Half of the participants received this condition, and half received the opposite instruction. Thus, the color and location of the stimulus defined two congruency conditions: congruent (i.e., stimulus presented at the same side of the screen as the hand assigned to respond) or incongruent (i.e., stimulus presented at side of the screen opposite to the hand assigned to respond). The shape of the stimuli (square or circle) indicated the type of response to perform (lifting or reaching) in Experiment 2. To maintain the same stimuli for both experiments, for the lifting action, stimuli were square shaped, whereas for the reaching action, stimuli were circle shaped. Half of the participants received this condition, and half received the opposite instruction. Notably, the shape of the stimuli (square or circle) was task irrelevant in Experiment 1. The order to perform the different blocks for lifting and reaching was counterbalanced. Each of the two simple blocks was composed of 144 trials. Participants performed 16 practice trials before each block; the practice trials were excluded from the data analysis. The experiment took about 20 min per participant.

Four dependent variables were recorded: mean reaction time (RT; i.e., the time elapsed between the presentation of the stimulus and the beginning of the motor action of the index finger), kinematic errors (KEs; i.e., trials in which participants began the action with the wrong hand), mean movement time (MT; or time from RT until the end of the reaching action), and movement errors (MEs; or trials in which the participant touched the wrong object or performed the movement badly and did not reach with accuracy). It should be noted that MT and MEs are not suitable to be contrasted between lifting and reaching. As has been pointed out in the Introduction, the Simon effect has an impact on reaction time (i.e., programming of movement) and rarely on posterior temporal latencies, such as movement time (De Jong et al., 1994; Rubichi et al., 2000; Rubichi and Pellicano, 2004; Iani et al., 2010). MT and MEs in reaching action were measured to rule out any unexpected Simon effect in this temporal segment.

Factorial repeated measures ANOVA was carried out on each dependent variable. For RT, the within-subjects factors were mean MT and KE rate, movement complexity (lifting vs. reaching), and congruence (incongruent vs. congruent). Partial eta square ($\eta_{p}^{2}$) was calculated as a measure of effect size. Planned comparisons were applied to the data to explain the interactions types among different levels of the studied variables. KE rate was not normally distributed; thus, a square root transformation was carried out, as in Moss and Muth (2011), to perform the respective ANOVA.

To analyze RT distribution according to the parameters of the activation–suppression model (Ridderinkhof, 2002a), delta-plots were constructed by following a path similar to that in Wylie et al. (2010) and Suarez et al. (2014). For the RT distribution, the magnitude of the Simon effect was plotted as a function of response time; RTs of correct trials of all subjects were rank ordered and then divided into four quartiles (bins).

Kinematic error distribution was measured by computing a conditional accuracy function, in which the accuracy rate is plotted as a function of RT. In this case, all incongruent trials (including KE) were considered for the construction of the bins, following the same path described above. Only incongruent trials were considered, as the activation–suppression model indicates that response activation can be obtained from the accuracy of faster incongruent trials. Thus, we analyzed the delta-plots of the Simon effect by following a procedure similar to that in Wylie et al. (2010). Factorial repeated measures ANOVA and planned comparisons were used to analyze the final slope segment (by connecting the third and fourth bins; Q3–Q4) of both the lifting and reaching actions. The slopes between bins were assumed to provide evidence of changes in the Simon effect along RT; according to the activation–suppression model (Ridderinkhof, 2002a), the effectiveness of the inhibition should be stronger at the end of the distribution (i.e., slower bins) (e.g., Wylie et al., 2010; Suarez et al., 2014). In addition, factorial repeated measures ANOVA and pairwise comparisons of means with Bonferroni adjustment were used to analyze the delta-plots of accuracy (i.e., KE rate) of incongruent trials, by comparing the within-subjects factor of bin (Q1, Q2) across all the conditions (i.e., bin × movement). Following the activation–suppression model (Ridderinkhof, 2002a), more errors were expected on the faster incongruent trials (i.e., faster bins) (e.g., Wylie et al., 2010). Data analysis was carried out with SPSS 21.0 (IBM Corporation, 2012).

### Results

In the data analysis, 3.93% of the trials were excluded, distributed among the following types: trials that included KEs (2.95%), trials that included MEs (0.39%), trials that were faster than 100 ms and slower than 2,000 ms in RT (0.37%), and trials that were slower than 1,200 ms in MT for the reaching action (0.22%). KEs and MEs were analyzed separately and excluded from the analyses of RT and MT.

#### Reaction Time

**FIGURE 2:**
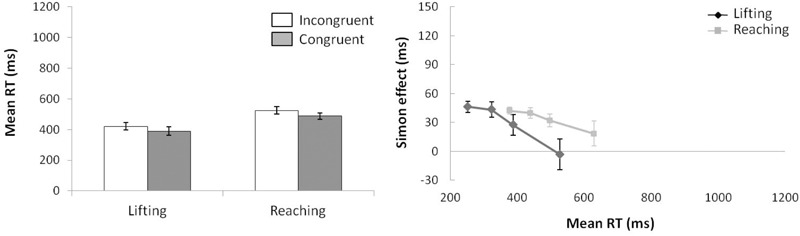
**(Left)** Interactions of movement × congruence, with respect to mean reaction time. **(Right)** Delta-plots showing Simon effect magnitude as a function of response speed (expressed in reaction time quartiles) for lifting and reaching actions in Experiment 1. Error bars represent the standard error of the mean.

For mean RT (see left panel of Figure 2) (ANOVA movement complexity × congruence), the main effect of movement complexity was significant, *F*(1,15) = 34.735, *p* < 0.001, $\eta_{p}^{2}$ = 0.698, with the lifting action 113 ms faster than the reaching action (372 vs. 485 ms, respectively). The main effect of congruence was also significant, *F*(1,15) = 30.627, *p* < 0.001, $\eta_{p}^{2}$ = 0.671, as incongruent trials were 31 ms longer than congruent trials (444 vs. 413 ms, respectively). Movement complexity × congruence interaction was not significant, *F*(1,15) = 0.562, *p* = 0.465, $\eta_{p}^{2}$ = 0.036, and the congruence effect did not differ significantly between movements (28 and 33 ms for lifting and reaching, respectively).

Regarding distributional analysis, the Simon effect delta-plots of both movements (lifting and reaching) are presented in the right panel of Figure 2. The main effect of movement complexity was not significant, *F*(1,15) = 0.894, *p* = 0.360, $\eta_{p}^{2}$ = 0.056, with similar slopes for lifting and reaching (*m* = -0.167, *m* = -0.055, respectively).

#### Movement Time for Reaching

For MT (congruence), the main effect of congruence, *F*(1,15) = 0.565, *p* = 0.464, $\eta_{p}^{2}$ = 0.036, was not significant (309 and 306 ms for incongruent and congruent, respectively).

#### Kinematic Errors

**FIGURE 3:**
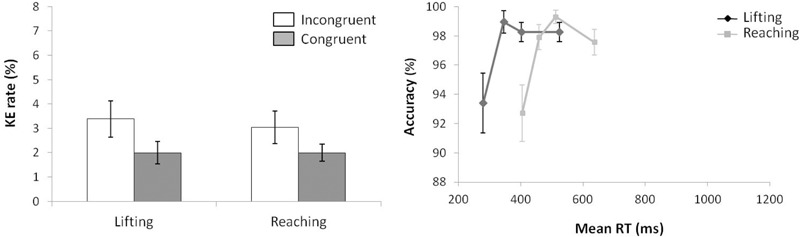
**(Left)** Interactions of movement × congruence with respect to kinematic error rate. **(Right)** Delta-plots showing conditional accuracy functions for incongruent trials as a function of response speed (expressed in reaction time quartiles) for lifting action (gray square) and reaching action (black circle) in Experiment 1. Error bars represent the standard error of the mean.

According to KE rate analysis (see left panel of Figure 3) for the next ANOVA (movement complexity × congruence), the main effects of movement complexity and congruence were not significant, (*F*(1,15) = 0.077, *p* = 0.785, $\eta_{p}^{2}$ = 0.005; *F*(1,15) = 2.678, *p* = 0.123, $\eta_{p}^{2}$ = 0.152), and neither was the interaction between these factors, *F*(1,15) = 0.002, *p* = 0.963, $\eta_{p}^{2}$ < 0.000.

Regarding distributional analysis, the right panel of Figure 3 shows the delta-plots of accuracy for incongruent trials of both actions (lifting and reaching). As previously pointed out, the two faster bins (Q1, Q2) were compared per condition (ANOVA movement complexity × bin). The results showed no main effect of movement, *F*(1,15) = 2.389, *p* = 0.143, $\eta_{p}^{2}$ = 0.137. However, the main effect of bin was significant, *F*(1,15) = 7.958, *p* = 0.013, $\eta_{p}^{2}$ = 0.347, presenting the first bin as having more KEs than the second bin (4.7% vs. 3.8%, respectively). The interaction of movement complexity × bin was not significant, *F*(1,15) = 0.384, *p* = 0.545, $\eta_{p}^{2}$ = 0.025.

#### Movement Errors for Reaching

For MEs, the main effect of congruence, *F*(1,15) = 0.008, *p* = 929, $\eta_{p}^{2}$ = 0.001, was not significant (0.61% vs. 0.52%, for incongruent and congruent trials, respectively).

## Experiment 2: Active-Movement Type Selection

### Methods

#### Participants

The experiment recruited 16 right-handed participants (seven males; mean age 21.37 ± 2.68 years) with normal or corrected-to-normal vision.

#### Apparatus, Stimuli, and Procedure

Apparatus, stimuli, procedure, and design were the same as those in Experiment 1, except for the following: The color of the stimulus defined the congruency of the response; the shape of the stimuli (square or circle) indicated the type of response to perform (lifting or reaching). For the lifting action, stimuli were square shaped, whereas for the reaching action, stimuli were circle shaped. As in Experiment 1, half of the participants received this condition, and half received the opposite instruction. This block was composed of 288 trials. Reaching and lifting were performed randomly. Before the experiment, participants performed 16 practice trials, which were excluded from the analysis. The duration of the experiment was about 20 min per participant. Data analyses were the same as those in Experiment 1.

### Results

In the data analysis, 4.08% of the trials were excluded, divided among the following types: trials that included KEs (2.97%), trials that included MEs (0.35%), trials that were faster than 100 ms and slower than 2,000 ms in RT (0.48%), and trials that were slower than 1,200 ms in MT for the reaching action (0.28%).

#### Reaction Time

**FIGURE 4:**
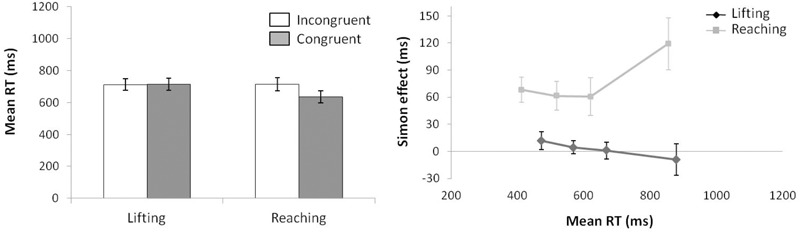
**(Left)** Interactions of movement × congruence, with respect to mean reaction time. **(Right)** Delta-plots showing Simon effect magnitude as a function of response speed (expressed in reaction time quartiles) for lifting and reaching actions in Experiment 2. Error bars represent the standard error of the mean.

For mean RT (see left panel of Figure 4) (ANOVA movement complexity × congruence), the main effect of movement complexity was significant, *F*(1,15) = 5.074, *p* = 0.0397, $\eta_{p}^{2}$ = 0.230, with the lifting action following an opposite trend compared with Experiment 1, being 46 ms slower than the reaching action (647 vs. 601 ms, respectively). The main effect of congruence was also significant, *F*(1,15) = 11.701, *p* = 0.004, $\eta_{p}^{2}$ = 0.438, as incongruent trials were 40 ms longer than congruent trials (644 vs. 604 ms, respectively). There was a significant first order interaction in movement complexity × congruence, *F*(1,15) = 30.643, *p* < 0.001, $\eta_{p}^{2}$ = 0.671. Planned comparisons showed that the effect of congruence differed significantly between movements without a congruence effect for lifting (2 ms; *p* = 0.827; 648 and 646 ms for incongruent and congruent trials, respectively) and a congruence effect for reaching (77 ms; *p* < 0.001; 640 and 563 ms, for incongruent and congruent trials, respectively).

The Simon effects delta-plots of both movements (lifting and reaching) are presented in the right panel of Figure 4. The main effect of movement complexity was significant, *F*(1,15) = 12.719, *p* = 0.003, $\eta_{p}^{2}$ = 0.459. The reaching action presented a positive-going slope (*m* = 0.245) that differed significantly from the negative-going slope of the lifting action (*m* = -0.080).

#### Movement Time for Reaching

For MT (congruence), the main effect of congruence was not significant, *F*(1,15) = 0.739, *p* = 0.404, $\eta_{p}^{2}$ = 0.047 (332 and 328 ms for incongruent and congruent trials, respectively).

#### Kinematic Errors

**FIGURE 5:**
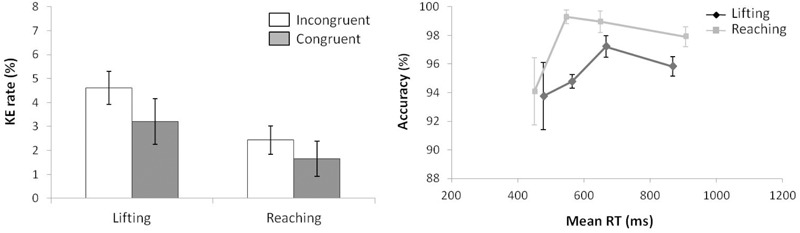
**(Left)** Interactions of movement × congruence with respect to kinematic error rate. **(Right)** Delta-plots showing conditional accuracy functions for incongruent trials as a function of response speed (expressed in reaction time quartiles) for lifting action (gray square) and reaching action (black circle) in Experiment 2. Error bars represent the standard error of the mean.

According to KE rate analysis (see left panel of Figure 5) for the next ANOVA (movement complexity × congruence), the main effect of movement complexity was significant, *F*(1,15) = 8.110, *p* = 0.012, $\eta_{p}^{2}$ = 0.351, as lifting action presented a larger KE rate than reaching (3.9 and 2.04, respectively.) A main effect of congruence was marginally significant, *F*(1,15) = 3.843, *p* = 0.069, $\eta_{p}^{2}$ = 0.204, as incongruent trials presented a larger KE rate than congruent ones (3.51% vs. 2.43%, respectively). The interaction of movement complexity × congruence was not significant, *F*(1,15) = 0.210, *p* = 0.654, $\eta_{p}^{2}$ = 0.014.

The right panel of Figure 5 shows the delta-plots of accuracy for incongruent trials of both lifting and reaching actions. Two faster bins (Q1, Q2) were compared per condition (ANOVA movement complexity × bin). The main effect of movement was marginally significant, *F*(1,15) = 3.747, *p* = 0.072, $\eta_{p}^{2}$ = 0.200, presenting the lifting action as having more KEs than the reaching action (4.6% vs. 2.4%, respectively). The main effect of bin was not significant, *F*(1,15) = 1.524, *p* = 0.236, $\eta_{p}^{2}$ = 0.092. The interaction of movement complexity × bin was significant, *F*(1,15) = 5.593, *p* = 0.032, $\eta_{p}^{2}$ = 0.272. The reaching and lifting actions differed significantly between bins. Differences were located in the second bin (5.2% vs. 0.6% for lifting and reaching, respectively; *p* = 0.004), but no differences per movement were found in the first bin (6.25% vs. 5.90% for lifting and reaching, respectively; *p* = 0.863).

#### Movement Errors for Reaching

The main effect of congruence for MEs was not significant, *F*(1,15) = 0.030, *p* = 0.865, $\eta_{p}^{2}$ = 0.002 (0.47% for both incongruent and congruent trials).

### Discussion

In Experiments 1 and 2, we aimed to determine whether response inhibition shows the same degree of effectiveness as a function of movement complexity and movement type selection. Thus, two components of response inhibition were examined following the activation–suppression model (Ridderinkhof et al., 2002a): activation of the preponderant responses (represented by error) and its subsequent response suppression (represented by magnitude of Simon effect). We hypothesized different magnitudes of the Simon effect with respect to the movement complexity of the actions: complex movement would show a smaller Simon effect (reflected in a more negative-going slope in the delta-plots) owing to higher error rates and slower latencies. The hypothesized differences could be more pronounced when action selection is required (active-movement type selection) because the experiment involved the extra process of action selection with subsequent longer latencies and higher error rate. Together, these hypothesized results would show that complex movements are inhibited more effectively in line with previous research (e.g., Greenhouse et al., 2015). The results partially corroborated these hypotheses.

Regarding our hypothesis on different activation and suppression as a function of movement complexity (Experiment 1), we found faster latencies for lifting actions compared with reaching actions. However, this phenomenon does not reflect different suppression effectiveness (i.e., different magnitudes for the Simon effect) between movements. This finding was corroborated by the delta-plots of the Simon effect, in which both motor actions showed the same pattern of negative-going slopes at the final segment, thereby reflecting effective response suppression built through time (Ridderinkhof et al., 2005; Suarez et al., 2014). In addition, no more KEs (which reflect activation of the preponderant responses) were found between movements, although the movement complexity between them was different. In short, Experiment 1 did not show different effectiveness levels of response suppression as a function of movement complexity, even when the lifting action was faster than the reaching action. In Experiment 2, active-movement type selection was measured compared with no active-movement type selection (Experiment 1). In contrast to the no active-movement type selection, the lifting action was strongly activated compared with the reaching action (more KE, especially in the first bins), with a subsequent more prominent suppression (smaller size of Simon effect and a more negative-going slope). This outcome corresponds with the idea that inhibitory control increases after errors in the Simon task, as a mechanism of cognitive control (i.e., reduction of impulse error responses). For reaching, movement did not evidence a decrease of the Simon effect but an increase in time (positive-going slope). Thus, and taking into account the results of Experiments 1 and 2, movement complexity *per se* did not impact the effectiveness of response suppression, even when it slowed down the response times. Nonetheless, the different effectiveness levels of response suppression in active-movement type selection lead to the conclusion that the inclusion of an additional mental operation, like action selection (i.e., movement selected to respond), drastically impacts this effectiveness.

The activation–suppression model could explain the results at least partially. We hypothesized that Simon effect should be smaller for the more complex movements because these movements take more planning and time, as well as leave more time for suppression of the incorrect responses. However, in movement complexity manipulation, there is no difference between movements with respect to magnitude in Simon effect. Thus, time *per se* seems to be an irrelevant condition to produce different magnitudes in Simon effect. Meanwhile, reaching action did not present more KEs. Thus, the suppression of incorrect response would not be necessary to reduce impulsive error responses, and consequently, the reaching actions would not present a reduction of Simon effect (i.e., more effective response suppression). This finding highlights the idea that response suppression is an act of cognitive control to minimize errors (Ridderinkhof et al., 2002b). Similar ideas have been proposed by conflict monitoring theory (Botvinick et al., 2001), in which the anterior cingulate cortex (ACC) monitors the occurrence of conflict in information processing. Upon the detection of conflict, this mechanism triggers similar strategic adjustment, as in activation–suppression model, to allow more efficient performance in the future and minimize error. A number of authors have postulated that suppression of the automatic route in Simon tasks is one of the mechanisms of online control in conflict monitoring theory (Stürmer et al., 2002). Reports have shown an increase in ACC activation after the commission of errors, and subsequently, the next trial displays a small Stroop interference (Botvinick et al., 2001). Thus, in movement complexity manipulation, a similar suppression mechanism would be involved owing to the absence of differences in error rates, which lead to similar magnitudes of Simon effects between reaching and lifting actions. The present results in movement complexity manipulation confirm previous findings on the absence of effect of movement complexity in task performance and inhibition (e.g., Pratt et al., 2014). Regarding the Simon effect, research on how motor manipulations impact its magnitude remains scarce. Mordkoff and Hazeltine (2011) compared responses with different motor complexity levels (button press vs. joystick movement); they did not find differences in the magnitude of the Simon effect between the two motor responses, and possible implications arising from this absence of differences were not discussed. Buetti and Kerzel (2010) studied how different motor responses (reaching and lifting) and types of eye movement (spontaneous, fixed, and saccade) affected response selection in the Simon task. Nevertheless, results were not discussed in terms of differences in inhibitory control as a function of motor complexity, as we propose in the present study. Other studies (e.g., Stoffels et al., 1989; Hommel, 1994a,b; Stoffels, 1996) have found that the magnitude of the Simon effect is occasionally smaller in more difficult tasks in terms of perceptual manipulations. A factor to explain the significant differences between magnitudes in Simon effects in different movements could be the differences in error rate for more difficult conditions. For example, Hommel (1994b) found higher error rates in high perceptual discriminability conditions where the Simon effect was smaller compared with low discriminability conditions. Accordingly, null Simon effects and relative weakness in the error rates were found to manipulate longer delays between spatial cue and target (Hübner and Mishra, 2013). In Mordkoff and Hazeltine (2011), in which no differences in magnitudes of Simon effect were found between movements, errors were uncommon and not subjected to formal analysis. Thus, the similarities in error rate could be translated in a similar and subsequent suppression between conditions. Supporting this logic, lifting action has a higher rate of error compared with reaching action in active-movement type selection condition, which leads to more effective suppression (lesser magnitude of Simon effect) in this condition, compared with reaching. This finding supports previous studies that suggested that the Simon effect could be influenced by task requirements (e.g., Wascher et al., 2001; Wiegand and Wascher, 2007; Buetti and Kerzel, 2008).

However, important questions remain unclear. Why was the lifting action more effectively inhibited than the reaching action in Experiment 2 (i.e., a lesser-magnitude Simon effect for lifting)? Why were the latency patterns found within the reaching and lifting movements of each experiment the reverse of each other?

The response may lie in the top-down strategy, necessary to inhibit strongly the action most that is prone to develop kinematic error during active-movement type selection and then minimize errors. The control necessary to stop the lifting action once it is started, in the case of an error, must be greater than that necessary to stop the reaching action, as the lifting movement is more automatic to execute. Thus, when this movement is being executed, the response system may be delayed (for congruent and incongruent trials) to enhance its chances of being able to inhibit the response. The lifting action in the active-movement type selection condition might have been strategically inhibited to avoid an excess of kinematic errors. Subsequently, the suppression may have prevented the decrease of the Simon effect with time in the lifting action. This idea is reinforced by the data from KEs in this condition. Moreover, we observed that the lifting action presented more errors than the reaching action in the second quartile within the active-movement type selection condition. Thus, the lifting movement exhibited a sustained error rate along the time distribution longer than the reaching action, as this action is more automatic and, therefore, more prone to impulsive responses that result in errors (in contrast, this result was not found in the no active-movement type selection). Similar results were found by Logan and Cowan (1984) and De Jong et al. (1995) in their studies of the selective-stop condition inside the Go/No-go paradigm. In the testing of this condition, the stop signal required participants to inhibit responses performed with one hand, referred to as the critical hand, but not those made with the other, the non-critical hand. Participants tended to delay selectively responses made with the critical hand to improve the inhibition of that hand’s response when required. In addition, similar enhanced strategic effects were found when stopping in more complex tasks compared with easy ones (Wessel and Aron, 2014). Several brain imaging studies have likewise shown that the stopping network can be recruited proactively to increase the chance of successful stopping (Wessel et al., 2012; Zandbelt et al., 2013). Further, this top-down strategy is consistent with the idea that response suppression is an act of cognitive control to minimize errors (Botvinick et al., 2001; Ridderinkhof et al., 2002a,b), and with previous findings that response inhibition is flexible and could be strategically inhibited to control the error rate in task switching contexts (Hübner and Druey, 2006; Marí-Beffa et al., 2012). Complementarily, our results could suggest that the activation of the action selection process causes cognitive resources to be split based on the aforementioned top-down strategy, more concretely allocating the majority of the resources to the most automatic action (i.e., lifting action). Notably, subjects’ resources are limited according to the central resources/capacity theories (Kahneman, 1973). Thus, the inhibition of lifting and reaching movements must be interpreted in conjunction. For example, the more effective suppression of lifting is allocated in the fourth quartile, accompanied by the less effective suppression of reaching that accounts for the strategic allocation of the majority of resources to the lifting action.

However, one crucial aspect must be taken into account as a possible explicatory factor for more effective response inhibition in the lifting action when action selection is activated (i.e., active-movement type selection: A different group of participants took part in each experiment. This setup presents advantages and disadvantages. The decisive advantage is the simpler design for statistical analysis, which simplifies the comprehension of the current research. Meanwhile, there are some important disadvantages. First, the individual differences could have caused the different patterns in movement type selection conditions between experiments. A benefit of using repeated-measures (using the same participants for both conditions in movement type selection) would allow exclusion of the effects of individual differences that could occur if two different groups of people were used (Howitt and Cramer, 2011). Second, there is not a direct statistical comparison between movement type selection conditions. The aforementioned disadvantages, and especially, the novelty of our findings, compelled us to conduct a new experiment in which the same participants performed all the experimental conditions (Experiment 3). The data were analyzed with a repeated-measures design.

## Experiment 3: Direct Interaction Between Movement Type Selection Conditions

### Methods

#### Participants

The sample comprised 16 right-handed participants (seven men, mean age = 20.31 ± 2.15 years).

#### Apparatus, Stimuli, and Procedure

The apparatus, procedure, and design were the same as in Experiments 1 and 2, except for the following changes. Experiment 3 comprised three experimental blocks, namely, responding by lifting in one block, reaching in another block, and reaching and lifting randomly in the third block. Blocks were balanced across participants in a Latin-square order. A factorial repeated measure analysis of variance (ANOVA) was carried out on each dependent variable (mean RT, mean MT, and KE rate) in the next within-subjects factors; movement type selection with two levels, no active-movement type selection vs. active-movement type selection, where reaching and lifting are performed in different blocks or in the same block, respectively, movement complexity with two levels (lifting vs. reaching) and congruence (incongruent vs. congruent).

### Results

In the data analysis, 4.47% of the trials were excluded and divided among the following types: those that included kinematic errors (3.17%), those that included movement errors (0.77%), those that were faster than 100 ms and slower than 2,000 ms in RT (0.34%), and, finally, those that were slower than 1,200 ms in MT for the reaching action (0.19%).

#### Reaction Time

**FIGURE 6:**
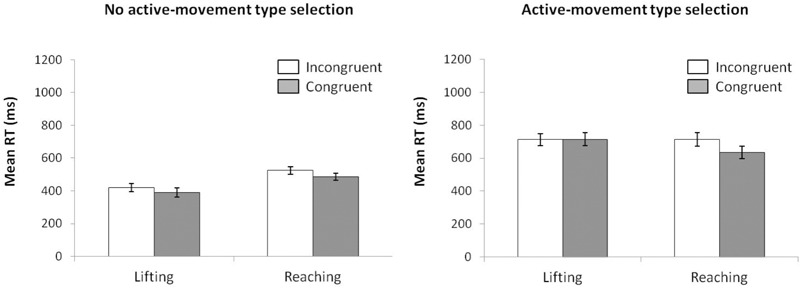
Interactions of movement type selection × movement complexity × congruence, within the no active-movement type selection condition **(Left)** and active-movement type selection **(Right)**, with respect to mean reaction time in Experiment 3. Error bars represent the standard error of the mean.

For mean RT (see Figure 6) (ANOVA movement type selection × movement complexity × congruence), the main effects of movement type selection, *F*(1,15) = 110.15, *p* < 0.001, $\eta_{p}^{2}$ = 0.880, movement complexity, *F*(1,15) = 5.027, *p* = 0.040, $\eta_{p}^{2}$ = 0.252, and congruence, *F*(1,15) = 36.784, *p* < 0.001, $\eta_{p}^{2}$ = 0.711, were significant. There first order interaction of movement type selection × movement complexity, *F*(1,15) = 81.606, *p* < 0.001, $\eta_{p}^{2}$ = 0.844, was significant. Within the no active-movement type selection condition, lifting was 101 ms faster than reaching, *p* < 0.001, whereas a reverse pattern was found within the active-movement type selection condition, with the reaching action being 39 ms faster than the lifting action, *p* = 0.042. The interaction of movement type selection × congruence did not reach significance, *F*(1,15) = 0.133, *p* = 0.721, $\eta_{p}^{2}$ = 0.008. The interaction of movement complexity × congruence was significant, *F*(1,15) = 18.545, *p* < 0.001, $\eta_{p}^{2}$ = 0.483; this interaction was mediated by movement type selection (second order interaction of movement type selection × movement complexity × congruence; *F*(1,15) = 14.108, *p* = 0.002, $\eta_{p}^{2}$ = 0.483). Planned comparisons showed that for no active-movement type selection, there was no interaction found between movement complexity and congruence (*p* = 0.346), whereas for active-movement type selection, such interaction was significant (*p* = 0.001). Thus, for no active-movement type selection, the congruence effect did not differ between movements (for the lifting action, 30 ms, *p* = 0.001; reaching action, 38 ms, *p* = 0.002). Regarding the active-movement type selection, the congruence effect differed significantly between movements without a congruence effect for lifting actions (-3 ms, *p* = 0.697) and a congruence effect for reaching actions (79 ms, *p* < 0.001).

**FIGURE 7:**
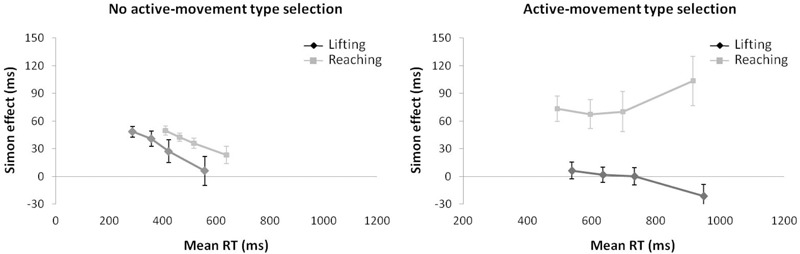
Delta-plots showing Simon effect magnitudes as a function of response speed (expressed in reaction time quartiles) for lifting and reaching actions within the no active-movement type selection condition (left side) and active-movement type selection (right side) in Experiment 3. Error bars represent the standard error of the mean.

Regarding distributional analysis, the Simon effect’s delta-plots of both movements (lifting and reaching) for ANOVA movement type selection × movement complexity (see Figure 7), the main effect of movement type selection was marginally significant, *F*(1,15) = 3.673, *p* = 0.075, $\eta_{p}^{2}$ = 0.196. The main effect of movement complexity was significant, *F*(1,15) = 5.785, *p* = 0.029, $\eta_{p}^{2}$ = 0.283. The first order interaction of movement type selection × movement complexity was not significant, *F*(1,15) = 1.583, *p* = 0.227, $\eta_{p}^{2}$ = 0.093. Nevertheless, planned comparisons showed significant differences. Within the no active-movement type selection condition, both the lifting slope (*m* = -0.183) and the reaching slope (*m* = -0.097) were negative-going and did not differ significantly, *p* = 0.561. In contrast, within the active-movement type selection condition, the delta-plot showed evidence of different patterns according to each movement. The reaching action presented a positive-going slope (*m* = 0.162) that differed significantly from the negative-going slope of the lifting action (*m* = -0.137, *p* = 0.002).

#### Movement Time for Reaching

For MT (movement type selection × congruence), the main effects of movement type selection, *F*(1,15) = 2.043, *p* = 0.173, $\eta_{p}^{2}$ = 0.127, and congruence, *F*(1,15) = 0.295, *p* = 0.594, $\eta_{p}^{2}$ = 0.057, were not significant; the interaction between these factors, *F*(1,15) = 1.002, *p* = 0.334, $\eta_{p}^{2}$ = 0.024, was also not significant.

#### Kinematic Errors

**FIGURE 8:**
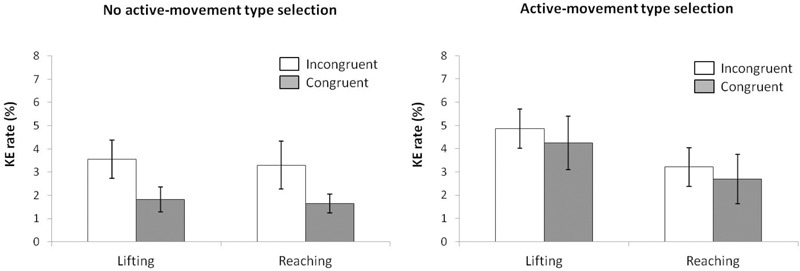
Interactions of movement × congruence within the no active-movement type selection condition **(Left)** and active-movement type selection **(Right)**, with respect to rate of kinematic errors in Experiment 3. Error bars represent the standard error of the mean.

According to the KE rate analysis for the next ANOVA (movement type selection × movement complexity × congruence, see Figure 8), the main effect of movement type selection was significant, *F*(1,15) = 88.214, *p* < 0.001, $\eta_{p}^{2}$ = 0.088, with a larger KE rate for active-movement type selection (3.75%) vs. no active-movement type selection (2.58%). The main effect of movement complexity was significant, *F*(1,15) = 6.046, *p* = 0.027, $\eta_{p}^{2}$ = 0.230, being mediated by movement type selection (the interaction is described below). A main effect of congruence was found, *F*(1,15) = 6.898, *p* = 0.019, $\eta_{p}^{2}$ = 0.371. As pointed out previously, the interaction of movement type selection × movement complexity was significant, *F*(1,15) = 5.891, *p* = 0.028, $\eta_{p}^{2}$ = 0.337. Within the no active-movement type selection condition, there is no difference between movements’ KE rate (2.7% vs. 2.5% for the lifting and reaching actions, respectively, *p* = 0.849). In contrast, in active-movement type selection condition, there is a larger KE rate for the lifting action (4.6%) compared with the reaching action (2.9%), *p* = 0.005. In addition, the lifting action presented a larger KE rate in active-movement type selection condition (4.6%) compared with no active-movement type selection condition (2.7%), *p* < 0.001, without differences for reaching action between the movement type selection conditions (2.9% and 2.7%, respectively; *p* = 0.840). The interactions of movement type selection × congruence, *F*(1,15) = 0.379, *p* = 0.551, $\eta_{p}^{2}$ = 0.055, and movement complexity × congruence, *F*(1,15) = 0.899, *p* = 0.770, $\eta_{p}^{2}$ = 0.051, were not significant; the second order interaction of movement type selection × movement complexity × congruence, *F*(1,15) = 0.985, *p* = 0.758, $\eta_{p}^{2}$ = 0.001, was also not significant.

**FIGURE 9:**
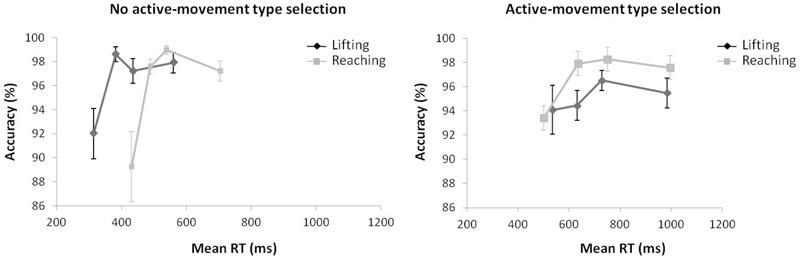
Delta-plots showing conditional accuracy functions for incongruent trials as a function of response speed (expressed in response time quartiles) for lifting action (gray square) and reaching action (black circle), within the no active-movement type selection **(Left)** and active-movement type selection **(Right)** in Experiment 3. Error bars represent the standard error of the mean.

Regarding distributional analysis, Figure 9 shows the delta-plots of accuracy for incongruent trials of both actions (lifting and reaching) within the movement type selection conditions. As pointed out previously, the two faster bins (Q1, Q2) were compared per condition (ANOVA movement type selection × movement Complexity × bin). There was no main effect of movement type selection, *F*(1,15) = 0.897, *p* = 0.359, $\eta_{p}^{2}$ = 0.033. The main effect of movement was not significant, *F*(1,15) = 3.595, *p* = 0.078, $\eta_{p}^{2}$ = 0.138. The main effect of bin was significant, *F*(1,15) = 7.159, *p* = 0.017, $\eta_{p}^{2}$ = 0.307, with the first bin having more kinematic errors than the second (7.5% vs. 2.6%, respectively). The first order interactions of movement type selection × movement complexity was not significant, *F*(1,15) = 0.243, *p* = 0.629, $\eta_{p}^{2}$ = 0.031. The movement type selection × bin interaction was marginally significant, *F*(1,15) = 4.210, *p* = 0.057, $\eta_{p}^{2}$ = 0.233, being mediated by movement complexity (the interaction is described below). The interaction of movement complexity × bin was not significant, *F*(1,15) = 1.465, *p* = 0.245, $\eta_{p}^{2}$ = 0.079. Nevertheless, the second order interaction of the movement type selection × movement complexity × bin was marginally significant, *F*(1,15) = 4.045, *p* = 0.062, $\eta_{p}^{2}$ = 0.201. Planned comparisons showed that for the no active-movement type selection condition, there was no interaction between movement complexity and bin (*p* = 0.420). In contrast, for the active-movement type selection condition, such interaction was significant (*p* = 0.046). Thus, within the no active-movement type selection condition, the reaching and lifting actions did not differ between bins. Regarding the active-movement type selection condition, the reaching and lifting actions differ significantly between bins. More concretely, these differences were in the second bin (5.6% vs. 2.1% for lifting and reaching, respectively; *p* = 0.028), but there was no difference per movement in the first bin (*p* = 0.757).

#### Movement Errors for Reaching

There was no main effect of congruence, *F*(1,15) = 0.115, *p* = 0.699, $\eta_{p}^{2}$ = 0.010,), and movement type selection. *F*(1,15) = 1.064, *p* = 0.319, $\eta_{p}^{2}$ = 0.066. The first order interaction of movement type selection × congruence was not significant, *F*(1,15) = 0.374, *p* = 0.550, $\eta_{p}^{2}$ = 0.024.

### Discussion

Overall, the results of Experiments 1 and 2 were replicated in Experiment 3. More importantly, the results of Experiment 3 confirmed the findings of both Experiments 1 and 2. Therefore, the individual differences between participants as an explanatory factor of the different patterns observed between movement type selection conditions were ruled out. Meanwhile, in Experiment 3, a statistical analysis was employed where movement type selection was measured directly to give consistency to the pattern of results obtained. In short, Experiment 3 confirmed that movement complexity *per se* did not affect response inhibition, which supports the findings of previous research (e.g., Pratt et al., 2014). However, when action selection is activated, the lifting action is strongly activated and suppressed. We argue that this could be due to the necessity of a top-down strategy to inhibit strongly the action that is most prone to develop a kinematic error during the active-movement type selection. In this way, errors in line with the activation–suppression model (and conflict monitoring theory) could be minimized. Moreover, the control of errors seems to have a determinant role in obtaining an interaction between response inhibition and motor complexity. However, it was found that slower reaction time is not crucial to produce a more effective suppression (a lesser magnitude of Simon effect), at least in movement complexity manipulation, which apparently nuances our initial predictions on the activation–suppression model.

## Experiment 4: Assessing Muscle Recruitment in Movement Complexity and Movement Type Selection

All above-mentioned experiments have confirmed that movement complexity *per se* did not affect response inhibition, which supports the findings of previous research (e.g., Pratt et al., 2014). However, when action selection was activated, the lifting action was strongly activated and suppressed. Thus, movement complexity would impact on response inhibition only in demanding situations where action selection is activated, with the subsequent necessity to inhibit the action that is most prone to develop a kinematic error. We carried out a new experiment to corroborate this hypothesis. In addition, we want to determine the extent to which the proposed movement complexity manipulation intended in terms of muscle recruitment (Henry and Rogers, 1960; Ma and Trombly, 2004), impacts on response inhibition. At this aim, lifting of the index and little fingers were compared since these actions are similar regarding their motor complexity. Both have a similar motor recruitment with extensor muscle (i.e., extensor digitorum muscle) being activated in both lifting actions. Moreover the extensor muscle of index fingers (i.e., extensor indicis muscle) and the extensor of the little fingers (i.e., extensor digiti minimi) are activated for index-lifting action and little finger-lifting action, respectively. It should be noted that middle and ring fingers are not suitable candidates to compare with index fingers because they do not have their own muscles to perform the lifting action. Thus, their lifting movements would have less involved muscles as compared to index-lifting movement. As far as movement complexity is concerned, we hypothesized that the equivalence in movement complexity would be reflected in similar sizes for Simon effect (reflected in similar going slopes in the delta-plots) owing to no differences in the error rate. Subsequently, and regarding to movement type selection, there was no reason to think that there would be any action most prone to develop kinematic error owing to similar motor complexity in more demanding situations (i.e., active-movement type selection). This could lead to no need for a top-down strategy to inhibit the action that is most prone to develop a kinematic error during the active-movement type selection, or in the same vein, a similar strategy to equally minimize the kinematic error for both actions. Thus, no differences between movement type selection conditions regarding motor inhibition were expected.

### Methods

#### Participants

The sample comprised 16 right-handed participants (eight men), mean age = 20.56 ± 2.22 years.

#### Apparatus, Stimuli, and Procedure

The apparatus, procedure, and design were the same as in Experiments 3, except for the following changes. Experiment 4 comprised three experimental blocks, namely, responding by lifting the index finger in one block, lifting the little finger in another block, and both lifting movements with both hands randomly in the third block. Thus, the variable movement complexity had the index-lifting vs. little finger-lifting levels. The button to respond with the index or little fingers when the actions were performed in isolation was placed to 33.5 cm from the center of the boxes where stimuli were presented. For the block where participants responded with index and little fingers randomly, two buttons were placed to 2.5 cm from the middle of button used in blocks where actions were performed in isolation.

### Results

In the data analysis, 3.55% of the trials were excluded and divided among the following types: those that included kinematic errors (3.33%), those that were faster than 100 ms and slower than 2,000 ms in RT (0.22%).

#### Reaction Time

**FIGURE 10:**
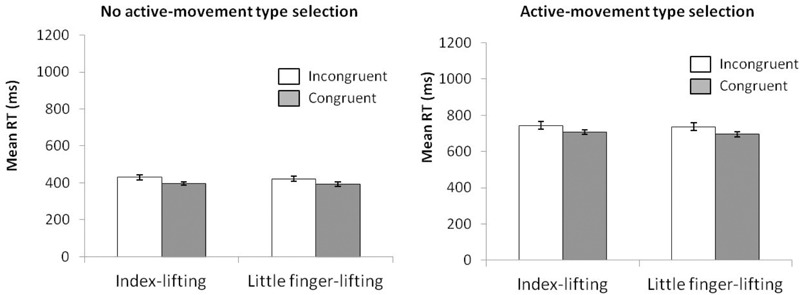
Interactions of movement type selection × movement complexity × congruence, within the no active-movement type selection condition **(Left)** and active-movement type selection **(Right)**, with respect to mean reaction time in Experiment 4. Error bars represent the standard error of the mean.

For mean RT (see Figure 10) (ANOVA movement type selection × movement complexity × congruence), the main effects of movement type selection, *F*(1,15) = 1157.7, *p* < 0.001, $\eta_{p}^{2}$ = 0.987, and congruence, *F*(1,15) = 23.078, *p* < 0.001, $\eta_{p}^{2}$ = 0.606, were significant. The main effect of movement complexity (562 and 569 ms for index and little finger lifting actions, respectively) was not significant, *F*(1,15) = 1.947, *p* = 0.183, $\eta_{p}^{2}$ = 0.115). The first order interactions did not reach significance: movement type selection × movement complexity, *F*(1,15) = 0.201, *p* = 0.660, $\eta_{p}^{2}$ = 0.013; movement type selection × congruence, *F*(1,15) = 1.027, *p* = 0.327, $\eta_{p}^{2}$ = 0.064; movement complexity × congruence, *F*(1,15) = 0.001, *p* = 0.980, $\eta_{p}^{2}$ < 0.001. The second order interaction of movement type selection × movement complexity × congruence neither reach significance; *F*(1,15) = 0.294, *p* = 0.596, $\eta_{p}^{2}$ = 0.019). Planned comparisons showed that for no active-movement type selection, the congruence effect did not differ between movements (*p* = 0.746). The Simon effect for the index-lifting action was 33 ms, and 27 ms for the little finger-lifting action, respectively. Similar results were found for active-movement type selection, the congruence effect did not differ between movements (*p* = 0.548). Simon effect for the index-lifting action was 36 ms and 39 ms for the little finger-lifting action.

**FIGURE 11:**
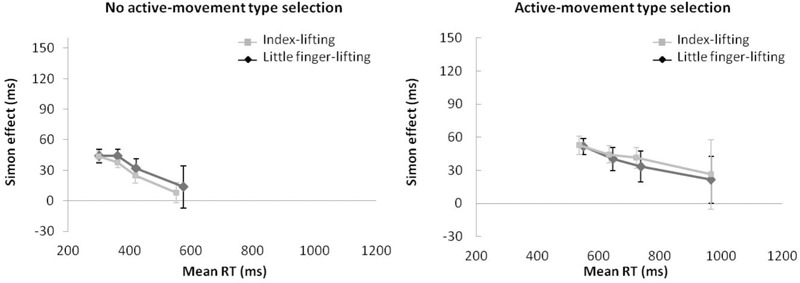
Delta-plots showing Simon effect magnitudes as a function of response speed (expressed in reaction time quartiles) for index-lifting and little finger-lifting actions within the no active-movement type selection condition **(Left)** and active-movement type selection **(Right)** in Experiment 4. Error bars represent the standard error of the mean.

Regarding distributional analysis, the Simon effect’s delta-plots of both lifting movements were compared (ANOVA movement type selection × movement complexity, see Figure 11). There were no main effects of movement type selection, *F*(1,15) = 0.916, *p* = 0.354, $\eta_{p}^{2}$ = 0.058, and movement complexity, *F*(1,15) = 0.057, *p* = 0.815, $\eta_{p}^{2}$ = 0.004. Neither the first order interaction of movement type selection × movement complexity, *F*(1,15) = 0.112, *p* = 0.742, $\eta_{p}^{2}$ = 0.007, was significant. Planned comparisons showed that for no active-movement type selection, both the index-lifting action slope (*m* = -0.114) and the little finger-lifting action slope (*m* = -0.161) were negative-going and did not differ significantly (*p* = 0.650). Similar results were found for active-movement type selection condition. Both the index-lifting action slope (*m* = -0.075) and the little finger-lifting action slope (*m* = -0.067) were negative-going and did not differ significantly (*p* = 0.956).

#### Kinematic Errors

**FIGURE 12:**
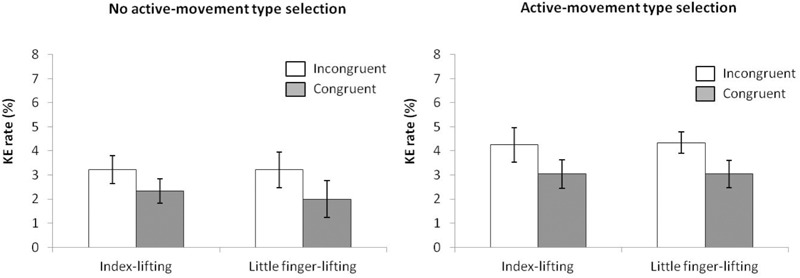
Interactions of movement × congruence within the no active-movement type selection condition **(Left)** and active-movement type selection **(Right)**, with respect to rate of kinematic errors in Experiment 4. Error bars represent the standard error of the mean.

According to the KE rate analysis (ANOVA movement type selection × movement complexity × congruence, see Figure 12), the main effects of movement type selection (2.9% vs. 3.7% for the no-active and active-movements type selection conditions, respectively), *F*(1,15) = 92,312, *p* < 0.001, $\eta_{p}^{2}$ = 0.999, and congruence (2.7% vs. 3.7% for congruent and incongruent trials, respectively), *F*(1,15) = 6.508, *p* = 0.022, $\eta_{p}^{2}$ = 0.303, were significant, whereas the main effect of movement complexity did not reach significance, *F*(1,15) = 0.025, *p* = 0.876, $\eta_{p}^{2}$ = 0.002, with similar error rates for both lifting actions (3.2% vs. 3.1% for index-lifting action and little finger lifting action, respectively). The first order interactions and the second order interaction were not significant, movement type selection × movement complexity, *F*(1,15) = 0.034, *p* = 0.856, $\eta_{p}^{2}$ = 0.002; movement type selection × congruence, *F*(1,15) < 0.001, *p* = 0.998, $\eta_{p}^{2}$ < 0.001; movement complexity × congruence, *F*(1,15) = 0.081, *p* = 0.780, $\eta_{p}^{2}$ = 0.005; movement type selection × movement complexity × congruence, *F*(1,15) = 0.529, *p* = 0.478, $\eta_{p}^{2}$ine-formula> = 0.034.

### Discussion

In Experiment 4, we aimed to determine the extent to which the muscle recruitment manipulation for movement complexity impacts on response inhibition. Thus, two components of response inhibition (activation and suppression), where examined for two actions with similar motor recruitment: index-finger lifting vs. little-finger lifting. We found comparable latencies for both lifting actions, with similar error rates and suppression effectiveness. Interestingly, this same pattern of results was found for both movement type selection conditions. Thus, it could be concluded that differences in movement complexity manipulation in terms of muscle recruitment have an impact on the effectiveness of motor inhibition. However, this impact is only restricted to really demanding situations, where the movement complexity manipulation causes more kinematic error and the subsequent necessity to inhibit the action that is most prone to develop a kinematic error. When movement complexity is similar, there is no difference in error rates suggesting that there is no need to prioritize one action over the other. This would produce a similar distribution of inhibition resources for both actions with a comparable strategy to minimize equally the kinematic error (or the absence of such strategy). Finally, it should be noted that the sole inclusion of an additional mental operation, such as action selection measured through movement type selection did not produce the aforementioned differences in motor inhibition. Thus, it would be necessary a higher error rate caused by movement complexity manipulation.

Future research should extend our results. For example, our manipulation of movement complexity was mainly related to different muscle recruitment. Thus, it would be necessary to study whether the current results could be generalized across other methods of varying motor complexity (i.e., different motor sequences, different trajectories, etc.). In addition, a study of switching cost (Monsell, 2003) and its derivatives (e.g., residual cost, switching cost, and implicit sequence learning) could contribute to provide additional evidence on strategic control in activation and suppression in response inhibition.

Finally, we considered different approaches to explain our results. Alternative models for Simon effect could explain, at least partially, by our results. Hommel (1994a,b) pointed out that the Simon effect could be reduced, and even eliminated, by the introduction of a manipulation that slows down the processing of the relevant stimulus information. This implies that the activation of the spatial stimulus code decays passively over time. Although previous studies (e.g., Servant et al., 2016; Salzer et al., 2017) have argued that the current evidence is inadequate to determine which model is more plausible (i.e., activation suppression model vs. passive decay hypothesis), these models are not mutually exclusive. Therefore, it is possible that both processes are involved in the Simon task; suppression could accelerate the decay of activation (Hübner and Mishra, 2013).

Another alternative explanation could be that reaching responses have a much higher congruence compared with incongruence with the stimuli. In the congruent condition, the stimulus was presented not only at the same side at which the response was required but also at the end point of the movement related to touching the stimulus. Consequently, the potential response conflict was much bigger for reaching responses than for lifting responses. In this line, Rubichi and Pellicano (2004) highlighted that the Simon effect in reaching action is considerably greater than in pressing action. They followed a different reasoning from inhibition in Ridderinkhof’s (2002a) activation–suppression model. More concretely, they suggested that for spatial tasks, the similarity between stimulus and response sets is greater for actions where the movement must be extended in space than for lateralized key-press responses. The greater Simon effect in reaching responses as compared to lifting in active-movement type selections could support this hypothesis.

A third alternative explanation could take into account that lifting response is the first part of reaching. Thus, the preparation of the reaching includes the preparation of lifting. Following this logic, it could be that the participants generally inhibit the less complex response (i.e., lifting) until they are sure that reaching is not required.

## Conclusion

We aimed to determine whether response inhibition shows the same or different degree of effectiveness as a function of two sources of motor complexity, namely, movement complexity and movement type selection. An activation–suppression model was used to measure activation of the preponderant responses and its subsequent suppression of impulsive motor responses in a Simon task. Interesting conclusions emerged from the pattern of data in this study. It seems that in our experiment, the manipulation of movement complexity *per se* did not impact on the effectiveness of motor inhibition. Nonetheless, the inclusion of an additional mental operation, such as action selection measured through movement type selection and an overall high error rate, has a remarkable impact on the suppression of impulsive motor responses, and, in turn, in the effectiveness of response suppression. In conclusion, these results occurred because of a top-down strategy to minimize error.

Our results shed light on how activation and suppression are involved in different motor tasks, highlighting that the locus of inhibition may be flexible, reflecting the behavioral requirements of the task (Tipper et al., 1992, 1994). This finding supports the previous studies, which have demonstrated that the mechanism underlying the strength of response inhibition in congruent task adjusts strategically to task demands (e.g., Hübner and Mishra, 2013). It also confirms several theories and models in cognitive control where conflict is the base for modulating control (e.g., Norman and Shallice, 1986; Botvinick et al., 2001).

## Author Contributions

GG-G, JA, and CB-S designed and performed the study, as well as analyzed the data. GG-G, JA, CB-S, LR, and GM wrote the manuscript.

## Conflict of Interest Statement

The authors declare that the research was conducted in the absence of any commercial or financial relationships that could be construed as a potential conflict of interest.
